# Complete genome analysis of *Tequatrovirus ufvareg1*, a *Tequatrovirus* species inhibiting *Escherichia coli* O157:H7

**DOI:** 10.3389/fcimb.2023.1178248

**Published:** 2023-05-16

**Authors:** Maryoris Elisa Soto Lopez, Marco Tulio Pardini Gontijo, Rodrigo Rezende Cardoso, Laís Silva Batalha, Monique Renon Eller, Denise Mara Soares Bazzolli, Pedro Marcus Pereira Vidigal, Regina Célia Santos Mendonça

**Affiliations:** ^1^ Departamento de Tecnologia de Alimentos, Universidade Federal de Viçosa, Viçosa, Minas Gerais, Brazil; ^2^ Departamento de Ingeniería de Alimentos, Universidad de Córdoba, Montería, Colombia; ^3^ Department of Molecular Genetics and Microbiology, Duke University Medical Center, Duke University, Durham, NC, United States; ^4^ Departamento de Microbiologia, Universidade Federal de Viçosa, Viçosa, Minas Gerais, Brazil; ^5^ Núcleo de Análise de Biomoléculas, Universidade Federal de Viçosa, Viçosa, Minas Gerais, Brazil

**Keywords:** whole genome sequencing (WGS), pan-genome, taxonomy, biocontrol, Enterohemorrhagic *Escherichia coli*

## Abstract

**Introduction:**

Bacteriophages infecting human pathogens have been considered potential biocontrol agents, and studying their genetic content is essential to their safe use in the food industry. *Tequatrovirus ufvareg1* is a bacteriophage named UFV-AREG1, isolated from cowshed wastewater and previously tested for its ability to inhibit *Escherichia coli* O157:H7.

**Methods:**

*T. ufvareg*1 was previously isolated using E. *coli* O157:H7 (ATCC 43895) as a bacterial host. The same strain was used for bacteriophage propagation and the one-step growth curve. The genome of the T. ufvareg1 was sequenced using 305 Illumina HiSeq, and the genome comparison was calculated by VIRIDIC and VIPTree.

**Results:**

Here, we characterize its genome and compare it to other *Tequatrovirus*. *T. ufvareg1* virions have an icosahedral head (114 x 86 nm) and a contracted tail (117 x 23 nm), with a latent period of 25 min, and an average burst size was 18 phage particles per infected *E. coli* cell. The genome of the bacteriophage *T. ufvareg1* contains 268 coding DNA sequences (CDS) and ten tRNA genes distributed in both negative and positive strains. *T. ufvareg1* genome also contains 40 promoters on its regulatory regions and two rho-independent terminators. *T. ufvareg1* shares an average intergenomic similarity (VIRIDC) of 88.77% and an average genomic similarity score (VipTree) of 88.91% with eight four reference genomes for *Tequatrovirus* available in the NCBI RefSeq database. The pan-genomic analysis confirmed the high conservation of *Tequatrovirus* genomes. Among all CDS annotated in the *T. ufvareg1* genome, there are 123 core genes, 38 softcore genes, 94 shell genes, and 13 cloud genes. None of 268 CDS was classified as being exclusive of *T. ufvareg1*.

**Conclusion:**

The results in this paper, combined with other previously published findings, indicate that *T. ufvareg1* bacteriophage is a potential candidate for food protection against *E. coli* O157:H7 in foods.

## Introduction

Enterohemorrhagic *Escherichia coli* (EHEC), a subgroup of Shiga toxin-producing *E. coli* (STEC), has emerged as one of the primary foodborne pathogens (Cooley et al., 2013). The infections’ symptoms vary from watery diarrhea to hemorrhagic colitis (HC) and, in more severe cases, hemolytic-uremic syndrome (HUS) (Gambushe et al., 2022). STEC pathogens belong to a wide range of serotypes, and *E. coli* O157:H7 is the most prevalent outbreak-associated serotype. Human infections are linked to consuming contaminated foods and water (Rani et al., 2021; Gambushe et al., 2022). *E. coli* O157:H7 has a low infectious dose (10 to 100 CFU.mL^-1^) due to its stress resistance mechanisms (Rahal et al., 2012), surviving in low-pH environments such as acidic foods. Acid resistance is crucial for the pathogen’s survival in the stomach’s acidic condition before reaching and colonizing the small intestines and/or colon (Leyer et al., 1995; Price et al., 2004). According to the Centers for Disease Control and Prevention (CDC), as of 2022, STEC was responsible for three outbreaks in the US. The number of cases reached 136, accounting for 63 hospitalizations. Similar outbreaks were reported yearly since 2006.

Classical preservation methods, including pasteurization, radiation, food preservatives (Lopez et al., 2018b; Enciso-Martínez et al., 2022), or lactic acid bacteria (Ren et al., 2019; Gontijo et al., 2020), have been used to control pathogenic bacteria in foods, such as *E. coli* O157:H7. Alternative methods are proposed to control biological contaminants in foods. For example, recent studies have suggested using bacteriophages to control bacteria for food production and processing, showing promising results (Moye et al., 2018; Endersen and Coffey, 2020).

Viruses are the most abundant entities on the planet, present in all living organisms’ ecosystems (Krishnamurthy et al., 2016; Koskella et al., 2022). Bacteriophage application as antibacterial agents in the food industry has some advantages over traditional methods: (i) phages are highly host-specific, infecting closely related species, species or even strains within a species (Peng and Yuan, 2018); (ii) selective toxicity, infection of humans and other eukaryotes by phages has been reported only in rare occasions (Lehti et al., 2017; Naureen et al., 2020); (iii) little or no influence on the gut microbiota (Galtier et al., 2016); (iv) phages do not alter the sensory properties of foods, once their genomes do not code substances that may change the color, flavor or texture of the foods (Moye et al., 2018); (v) phages are self-replicating and self-limiting if their bacterial host is present (Sillankorva et al., 2012); and (vi) the frequency of phage mutation is higher than that of bacteria, which diminishes the chances of bacterial resistance (Sabouri Ghannad and Mohammadi, 2012).

Bacteriophages have been evaluated for clinical use (Morello et al., 2011; Merabishvili et al., 2017; Zaki et al., 2023), agriculture (Czajkowski et al., 2017; Zaczek-Moczydłowska et al., 2020; Liu et al., 2022), and the food industry (Soffer et al., 2017). A few phage-based products have been approved by the Food and Drug Administration (FDA), ListShield (used in salami, sausage, basterami, seafood, food contact surfaces, and environments), EcoShield (applied to red meat parts and trim intended to be grounded) and SalmoFresh (applied to poultry, fish and shellfish, fresh and processed fruits, and vegetables), all produced by Intralytix, Inc. in the USA. LISTEX, produced by Food Safety in the Netherlands for use in ready-to-eat meat, fish, and cheese, and Agriphage, made by Omnilytics in the USA, for use in agriculture on fruits and vegetables (Jamal et al., 2019). However, more studies are being conducted for further approval and regulation. Phage vB-LmoM-SH3-3 was effective in reducing the *Listeria* spp. count in salmon meat, reducing by approximately 2.67 log CFU.mL^-1^ after 24 h of phage addition at 4°C and 4.14 log CFU.mL^-1^ after 48 h. Similar results were found for orange juice (Zhou et al., 2020). Encapsulated phages specific for *Salmonella* reduced the count of *Salmonella* Enteritidis and S. Typhimurium in 0.57 and 1.78 log CFU.cm^-2^ in meat and 0.86 and 1.2 log CFU.g^-1^, respectively, in sprout (Petsong et al., 2019). *Tequatrovirus ufvareg1*, in particular, has already been evaluated against *E. coli* O157:H7 in the biosanitization of cherry tomatoes (Lopez et al., 2018a). The treatment of cherry tomatoes inoculated with *E. coli* O157:H7 was reduced by 0.95 log CFU.g^-1^ after adding a cocktail of bacteriophages (*T. ufvareg1* included). This value was statistically similar to the decontamination treatment of tomatoes with the sodium dichloroisocyanurate (0.57 log CFU.g^-1^), hydrogen peroxide (0.98 log CFU.g^-1^), and peracetic acid (0.9 log CFU.g^-1^). *T. ufvareg1* encapsulated in alginate *via* microfluidics and applied in a propylene glycol gel showed efficiency in the reduction of *E. coli* O157:H7 count (reducing from 4 log CFU.g^-1^ to an undetectable count on the surface) at a similar level to alcohol 70% (Boggione et al., 2017). In addition, the bacteriophage UFV-AREG1 was encapsulated in several food hydrocolloids and remained viable when submitted to pH 2.5. Phage-loaded beads incubated in simulated intestinal fluid (pH 6.8) resulted in a 50% release of the phages in the first 5 min. These results also highlight the potential of phage UFV-AREG1 to control pathogens in livestock (Silva Batalha et al., 2021). Such reductions in foods or food-related environments could substantially decrease the potential risk of foodborne infections caused by *E. coli* O157:H7.

Phages specific to *E. coli* O157:H7 have previously been isolated in several niches (Lu and Breidt, 2015; Shahin et al., 2022). To expand our understanding of the *E. coli* O157:H7-specific *Tequatrovirus ufvareg1*, we present the analysis of its morphology, growth parameters, host range, and genomic features in detail. In addition, information about *T. ufvareg1* will be helpful in the development of phage control of multiple foodborne pathogens.

## Materials and methods

### Bacterial strains and culture conditions

The strain *Escherichia coli* O157:H7 (ATCC 43895) was used as the host for bacteriophage propagation and characterization. The strain was cultivated in Brain Heart Infusion Broth (HiMedia, Brazil) growth medium and incubated at 37°C. Bacteriophage UFV-AREG1 from *Tequatrovirus ufvareg1* specie was previously isolated and sequenced (Lopez et al., 2016). Bacteriophages were stocked and diluted in SM buffer (gelatin 0,01% [m/v] [Vetec, Brazil], Tris-HCl 50 mM [pH 7,5] [Sigma Aldrich, Brazil], NaCl 100 mM [Vetec, Brazil], MgSO_4_.7H_2_O 8 mM [Vetec, Brazil]).

### Electron microscopy

A bacteriophage suspension at 10^9^ PFU.mL^-1^ was centrifuged at 26,000 ×g for 60 min. The pellet was washed with an ammonium acetate solution (0.1 M) [Sigma Aldrich, Brazil] and centrifuged at 26,000 ×g for 60 min. The pellet was resuspended in distilled water and filtered through a cellulose acetate membrane (0.22 µm pore). A droplet of the suspension was deposited on the surface of an electron microscopy screen coated with formvar resin. Virions were negatively stained with uranyl acetate (2% w/v) [Sigma Aldrich, Brazil], and micrographs were taken under 80 kV transmission electron microscope Zeiss EM 109 at the *Núcleo de Microscopia e Microanálise* (NMM/UFV).

### One-step growth curve

The one-step growth experiment was performed following the procedure described previously (Lu and Breidt, 2015). The *E. coli* O157:H7 (ATCC 43895) culture was activated in BHI broth until the exponential growth phase. The cells were centrifuged at 10,000 ×g for 5 min, and a suspension was prepared in saline solution (0.85% w/v NaCl) [Vetec, Brazil] with turbidity equivalent to 0.5 in the McFarland scale (10^8^ CFU.mL^-1^). The bacterial suspension was 10-fold diluted, and 0.1 mL was added in 0.8 mL of BHI broth preheated to 37°C. A phage suspension was prepared in SM buffer (10^5^ PFU.mL^-1^), and 0.1 mL was added to the tube with the bacterial culture (MOI: 0.01), followed by incubation at 37°C for 10 min at 100 rpm. After incubation, the suspension was centrifuged at 13,000 ×g for 30 s, and the supernatant was removed to determine the titer of non-adsorbed bacteriophages. The precipitate with absorbed bacteria and bacteriophages was resuspended in 20 mL of BHI broth preheated to 37°C. Subsequently, 1 mL samples were filtered (0.22 μm) and collected every 5 min for 60 min. Phage count was made by the double layer plating technique, and the *T. ufvareg1* phage titer was determined on plaque forming units (PFU) plating ten-fold serial dilutions in SM buffer (Green et al., 2012). The latent period was defined as the time interval between the end of the adsorption and the beginning of the lysis, as indicated by the beginning of the period of increase in the bacteriophage titer. Burst size was calculated by dividing the final number of released bacteriophages by the number of infected bacteria. The number of infected bacteria was estimated through the difference between the initial number of bacteriophages and non-adsorbed bacteriophages.

### Sequencing of the *Tequatrovirus ufvareg1* genome

The DNA of the *T. ufvareg1* bacteriophage was extracted according to a previously described procedure (Green et al., 2012). A bacteriophage suspension of 1 mL was added to 20 µL of chloroform [Vetec, Brazil] and stirred for 10 min. After stirring, the suspension was centrifuged at 22,000 rpm for 20 min. The aqueous phase was added of DNAse [Thermo Scientific, Brazil] and RNAse [Thermo Scientific, Brazil] to a final concentration of 1 µg.mL^-1^ for 10 min at room temperature. Subsequently, 25 µL of proteinase K [Thermo Scientific, Brazil] was added to a final concentration of 1 mg.mL^-1^ and 0.9 µL of SDS [Sigma Aldrich, Brazil] for 10 min at room temperature. A first extraction was made using phenol-chloroform (1:1) [Vetec, Brazil] for 5 min. It proceeded to centrifuge at 12,000 rpm for 10 min, recovering the aqueous phase. In the second extraction, chloroform was added in the proportion (1:1). Afterwards, the samples were centrifuged at 12,000 rpm for 10 min at 4°C. The aqueous phase was recovered. Ammonium acetate was added at a final concentration of 3.5 M for DNA precipitation and isopropanol in the ratio (1:1), storing the samples at -20°C for approximately 4 h. After this procedure, the samples were centrifuged at 12,000 rpm for 30 min, discarded the supernatant, and dried at room temperature. To remove excess salt from the samples, 200 µL of 70% ethanol [Vetec, Brazil] was added by centrifuging at 12,000 rpm for 10 min, discarding the ethanol, and placing the pellet to dry at 37°C for 10 min. The pellet was reconstituted with 40 µL of ice-cold ultrapure water [Sigma Aldrich, Brazil] and homogenizing. Finally, the DNA extraction process was evaluated on 0.8% agarose gel [Vetec, Brazil] with a 50 ng.mL^-1^ lambda marker. The genome of the *T. ufvareg1* was sequenced using Illumina HiSeq by the company Macrogen (Seoul, Korea).

### *De novo* assembly and annotation of the *T. ufvareg1* genome

The quality of sequencing data was assessed using FASTQC version 0.11.9 (https://github.com/s-andrews/FastQC). Adapter sequences were detected and removed from sequencing data using the “auto-detection” setting of TrimGalore version 0.6.7 (Krueger et al., 2021). Then, paired reads were trimmed for quality and filtered for length using Trimmomatic version 0.39 (Bolger et al., 2014) by selecting the following parameters: HEADCROP:10, CROP:90, SLIDINGWINDOW:4:20, and MINLEN:50. The *de novo* assembly of the genome was performed using the method “careful” of SPAdes version 3.15.3 (Bankevich et al., 2012) and testing all odd k-mers between 21 and 89. Then, the paired reads were mapped in the scaffolds using the BWA-MEM algorithm of BWA version 0.7.17 (Li and Durbin, 2009), and the Sequence Alignment Map (SAM) files were converted to ordered Binary Alignment Map (BAM) format using Picard toolkit version 2.26.2 (https://github.com/broadinstitute/picard). The BAM files were processed by SSPACE version 3.0 (Boetzer et al., 2011), which merged the scaffolds into a single circular sequence (**Figure S1**).

Eighty-four genomes from species of the *Tequatrovirus* genus (taxonomy ID: 10663) were downloaded from NCBI Reference Sequence (RefSeq) database (https://www.ncbi.nlm.nih.gov/refseq/, accessed on August 29^th^, 2022) (**Table S1**) and used as references to predict the genes of the *T. ufvareg1* genome. The genes were predicted using Prokka version 1.14.6 (Seemann, 2014) by selecting the following parameters: compliant, kingdom: viruses, gcode: 11, cdsrnaolap, E-value: 1e−10, proteins: *Tequatrovirus* Refseq GenBank file. The genome was also inspected to identify putative promoters using the PhagePromoter (https://galaxy.bio.di.uminho.pt/) (Sampaio et al., 2019) by selecting the following parameters: threshold: 0.5, phage family: *Podoviridae*, host bacteria genus: *Escherichia coli*, and phage type: virulent. The Rho-independent terminators were identified using ARNold (Naville et al., 2011) (http://rssf.i2bc.paris-saclay.fr/toolbox/arnold/) and FinTerm (Solovyev and Salamov, 2011) by considering a free energy threshold value of -11 kcal/mol for stem-loop regions. The phage host range was previously assessed (Lopez et al., 2018a) and expanded using HostPhinder(Villarroel et al., 2016), and the results were compared to previously published data for *T. ufvareg1* bacteriophage(Lopez et al., 2018a). Antibiotic resistance genes were predicted using CARD (Alcock et al., 2023).

### Endolysin and holin screening

Endolysins and holins were screened using HmmerWeb version 2.41.2 (Potter et al., 2018) and the protein family database (Pfam), applying default parameters. Possible signal peptides and signal-arrest-release domains in endolysins were characterized previously (Gontijo et al., 2021b; Gontijo et al., 2022). Signal peptides were predicted using SignalP version 5.0 (Petersen et al., 2011) and PrediSi version 1.0 (Hiller et al., 2006) against the Gram-negative database. Transmembrane regions were predicted using SOSUI version 1.1 (Hirokawa et al., 1998), TMHMM version 2.0 (Krogh et al., 2001), Phobius version 1.01 (Käll et al., 2004), and Topcons version 1.0 (Hennerdal and Elofsson, 2011).

### Comparative genomics of *Tequatrovirus*

The Eighty-four *Tequatrovirus* genomes, downloaded from NCBI Reference Sequence (RefSeq) database (**Table S1**), were compared with the genome of *T. ufvareg1*. All the genomes were opened in the rIIA gene, which is the same point as the *Tequatrovirus T4* genome (NCBI accession NC_000866). A pairwise genomic similarity matrix was calculated by the VIRIDIC web server (http://rhea.icbm.uni-oldenburg.de/VIRIDIC/) (Moraru et al., 2020) as previously described (Hungaro et al., 2022), considering a genomic similarity threshold 95% for species assignment and 70% for the genus as that recommended by ICTV for virus taxonomy. In addition, a proteomic tree was calculated by the Viral Proteomic Tree (VIPTree) web server (https://www.genome.jp/viptree/) (Nishimura et al., 2017), with default parameters. The synteny shared among the *Tequatrovirus* genomes was evaluated by Clinker version 0.0.23 (Gilchrist and Chooi, 2021), with default parameters. The repertoire of genes shared among the Tequatrovirus genomes was predicted by Roary version 3.13.0 (Page et al., 2015), considering a minimum identity threshold of 80% to cluster the protein sequences in the pangenome and setting that the core genes are shared among all genomes.

## Results and discussion

### The *Tequatrovirus ufvareg1* bacteriophage

**Figure 1:**
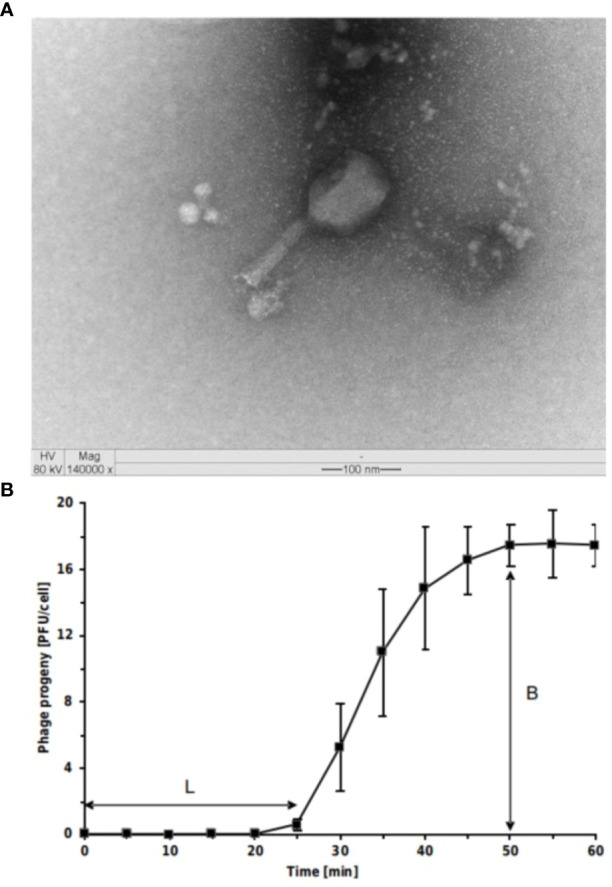
Virions and one-step growth curve of *Escherichia coli* O157:H7 phage *Tequatrovirus ufvareg1*. **(A)** the phage virion was negatively stained with uranyl acetate and observed through transmission electron microscopy (TEM) at × 140,000 magnification, scale bar = 100 nm. **(B)** The results of one-step growth curves of *T. ufvareg1* on *E coli* O157:H7 (ATCC 43895) are presented as the mean values ± SD from three independent experiments. L, latent period; B, burst size.

*T. ufvareg1* is a bacteriophage isolated from cowshed wastewater using *Escherichia coli* ATCC 11229 as the bacterial host (Lopez et al., 2016). A detailed characterization of this bacteriophage is presented in the following topics. Transmission Electron Microscopy analyses showed that *T. ufvareg1* virions have an icosahedral head (114 x 86 nm) and a contracted tail (117 x 23 nm) (**Figure 1A**). Therefore, *T. ufvareg1* belongs to *Caudoviricetes* class and shares a morphological similarity with the *Tequatrovirus* phages, such as *Tequatrovirus* hy01(Lee et al., 2016).

### Latent period and burst size of *T. ufvareg1*

To elucidate the ability of *T. ufvareg1* bacteriophage to lyse *E. coli* O157:H7 (ATCC 43895), the latent periods and burst size of the bacteriophage were determined using a one-step growth curve analysis (**Figure 1B**). The latent period of *T. ufvareg1* bacteriophage was 25 minutes, the complete infectious lasted 50 minutes, and the burst size after lysis of the *E. coli* host was about 18 PFU per infected cell. These results are similar to the one observed for *Tequatrovirus hy01* (latent period of 25 minutes and burst size of 25 bacteriophages per infected cell) (Lee et al., 2016). The burst size is an average of bacteriophages released by the bacterial cells in culture, but the capacity of virion production varies from cell to cell (Kannoly et al., 2022). Bacteriophages specific for *E. coli* O157:H7 have varying latent periods and burst sizes. Bacteriophage vB_Eco4M-7 has a latent period of 10 min, with a burst size of approximately 100 phages per cell (Necel et al., 2020). Bacteriophage Φ241 has a latent period of 15 min and a burst size of 53 phage particles per infected cell (Lu and Breidt, 2015), and bacteriophage SFP10 has a latent period of 25 min and releases about 100 new viral particles after the infectious cycle (Park et al., 2012). The burst size of *E. coli* O157:H7 bacteriophages varies substantially depending on the bacteriophage. From multiple isolates using the same host strains, it was previously observed a burst size ranging from 91 to 522 virion particles per infected cell (Montso et al., 2019). Generally, a phage with both a short latent period and a large burst size may have a selective advantage over competing phages and might be more effective in phage therapy (Park et al., 2012; Lu and Breidt, 2015; Amarillas et al., 2017). This result might indicate a need for bacteriophage combinations. In the case of *T. ufvareg1* bacteriophage, an effective reduction in bacterial count *in situ* has been observed in a bacteriophage cocktail (Lopez et al., 2018a).

### *T. ufvareg1* genome analysis

The complete nucleotide sequence of the genome of *Tequatrovirus ufvareg1* has been previously assembled using a reference-based approach and deposited in the GenBank database (https://www.ncbi.nlm.nih.gov/genbank/) under accession number KX009778.4 (Lopez et al., 2016). To understand the genomic organization of the new virus species, we updated this genome using a *de novo* assembly and deposited it in Genbank under accession KX009778.4. The *T. ufvareg1* has a linear dsDNA genome with 167,231 bp and a GC content of 35.35%, similar to what was observed for the *Tequatrovirus* reference genomes (average values: size = 167,831, GC content = 35.39%) (**Supplementary Material**, **Table S1**). The *T. ufvareg1* genome has a bidirectional organization with 278 genes corresponding to a gene density of 1.66 genes per 1,000 bp. Information about functional annotation of the coding DNA sequences (CDS) and their respective proteins is detailed in **Table S2**. Among the 268 predicted CDS, 113 encode hypothetical proteins (40.65%) that share similarities with other *Tequatrovirus* bacteriophages proteins and have no defined function in the replication and viral infection of *T. ufvareg1* (**Table S2**).

The search for consensus sequences of regulatory elements revealed the presence of 18 phage-specific promoters (score threshold = 0.5) and 325 host promoters (score threshold = 0.9) (**Table S3**) in the *T. ufvareg1* genome. In the positive strand, 13 promoters are found regulating mainly the late genes from the structural gene module. The remaining five promoters are located on the negative strand, including one regulating the gene that encodes the ModB ADP-ribosylase involved in regulating the replication cycle. The host promoters are distributed across the *T. ufvareg1* genome, with 108 in the positive strand and 217 in the negative strand. The presence of promoters is a common feature of viral genomes to start and control the gene transcription and consequent protein expression during their infection cycle (Eller et al., 2014; Sampaio et al., 2019). In addition, 20 rho-independent terminator sequences were found by both predictors in the *T. ufvareg1* genome, seven located at the positive and 13 at the negative strand (**Table S4**). As observed for the predicted promoters, the rho-independent terminators are found mainly immediately after the late genes from the structural gene module and after the genes involved in the preparation of the viral genome replication process (Bonocora et al., 2011; Pham et al., 2020). The second terminator is located after the gene encoding DNA polymerase, an essential enzyme for viral replication (Morcinek-Orłowska et al., 2022). The low number of rho-independent terminator sequences (20 terminators), concerning the large number of genes identified in the *T. ufvareg1* genome (278 genes), can be explained by the existence of another type of not conserved terminators, the modular structure of the genome of the phage or even the generation of polycistronic mRNAs (Wicke et al., 2021).

Furthermore, ten tRNA genes were found in the genome of the *T. ufvareg1* bacteriophage (**Tables S1**, **S5**), the same number observed for 24 of 84 *Tequatrovirus* reference genomes. However, the exact function of the tRNAs encoded by bacteriophages is unclear. These elements of the genome may be involved in adapting to the translation system of the bacterial host, to the demand for codon use pattern by the bacteriophage, or the rapid extinction of the specific translation of the host proteins after phage infection (Miller et al., 2003; Delesalle et al., 2016). A possible function of tRNAs in the phage genome may be to allow greater efficiency in translating specific unessential genes with different codon uses (Yang et al., 2021) or the ability of phages to growth in the host or to infect more hosts (Delesalle et al., 2016).

The 268 CDS predicted in the *T. ufvareg1* genome were classified into eight functional categories (“gene expression related proteins,” “metabolism-related proteins,” “cell wall lysis,” “structural proteins,” “direct lysis, host defense, and resistance-genes acquisition proteins,” “hypothetical proteins,” “homing endonucleases,” and “superinfection immunity protein”) (**Table S2**).

Thirty-four CDS distributed in the positive strand of the UFV-AREG1 bacteriophage genome (CDS numbers 153 to 178, 192 to 197, and 249 to 251) encode “structural proteins” of the virion, such as capsid, tail, and head proteins (**Table S2**). These CDS can be considered late genes and directly relate to the lysis processes after assembling the virus, DNA maturation, and viral particle release. Other 11 CDS that encode “structural proteins” are in the negative strand of the UFV-AREG1 genome (CDS numbers 27, 35, 39, 146, 148, 150, 152, 182, 183, 190, and 191), and they are probably expressed in parallel to the viral metabolism and replication (**Table S2**).

Seven CDS encode “direct lysis, host defense, and resistance-genes acquisition proteins,” and four ORFs encode “cell wall lysis” proteins (CDS 103, 120, 154, and 254), including one lysozyme encoded by CDS 120. The lysozyme encoded by CDS 120 is related to the bacteriophage infection process and the degradation of peptidoglycan to allow the entry of viral genetic material into the host. The lysozyme encoded by CDS 154 is an endolysin with the activity that degrades the peptidoglycan and acts together with holin (CDS 254) in the sequential events that lead to the programmed lysis of the host for the release of mature viral particles (Lu et al., 2019). Holin is responsible for permeabilizing the host cell membrane and signaling the exit of lysine to the periplasmic space for peptidoglycan cleavage, facilitating the release of the viral progeny (Wang et al., 2000; Saier and Reddy, 2015). Holin is strongly regulated by antiholin (Guo et al., 2018) encoded by CDS 103, located on the negative strand of the genome of the *T. ufvareg1* bacteriophage. The CDS classified as “direct lysis, host defense, and resistance-genes acquisition proteins” are related to the protection of replication processes, in general, to prevent the action by the host from interrupting the viral genome replication process, the assembly of the virus itself and the cell lysis.

In the negative strand of the *T. ufvareg1* genome, 102 CDS encode proteins responsible for bacteriophage replication classified as “gene expression-related proteins” or “metabolism-related proteins.” The *T. ufvareg1* bacteriophage genome opens with the CDS that encodes the protein rIIA, typical of bacteriophages with lytic activity. The genome of *Tequatrovirus* bacteriophages has its opening of the genome with the protein rIIA, and the evidence suggests that proteins of type rII are not directly related to the processes of inhibition of cell lysis. When they are absent, an alternative route for the cell lysis process depends on the presence of genes from some possible prophages (Miller et al., 2003; Abedon, 2019). The rIIA protein can be crucial in viral replication if it is found in clusters carrying different enzymes that synthesize deoxyribonucleotides and are associated with both the DNA and membrane of the host, as seen in the representative scheme of the *T. ufvareg1* bacteriophage genome. In addition, it might be required to maintain the integrity of the host membrane during infection and replication (Ennis and Kievitt, 1973). Other essential proteins in the DNA replication processes of *T. ufvareg1* are the exonuclease (encoded by CDS 13), ModA RNA polymerase ADP-ribosylase (CDS 19), UvsX RecA-like recombination protein (CDS 40), DNA polymerase (CDS 47), UvsW helicase (CDS 184 and 186), DNA ligase (CDS 202) and DNA topoisomerase II (CDS 265). Regarding DNA helicases (CDS 184 and 186), it is interesting to note that they are found on the positive strand of the phage genome. In contrast, the other CDS related to viral replication is in the negative strand.

Three CDS encode proteins related to “superinfection immunity protein” (CDS 37, 44, and 78). CDS 44 encodes an “immunity to a superinfection membrane protein,” which prevents the phage genome from being ejected from the cell cytoplasm (Berngruber et al., 2010; Egido et al., 2022). Likewise, this protein is responsible for preventing infection of the bacterium by other phages of the same family and recognizing the cell receptors on the bacterial surface (Abedon, 2015). In addition, one CDS encodes a “homing endonucleases” in the UFV-AREG1 bacteriophage genome (CDS 244), which might suggest the presence of introns in the genome of this phage, which will be confirmed in the future. The *T. ufvareg1* phage contains five CDS classified as “metabolism-related proteins” that encode recombination endonucleases (CDS 55, 58, 77, 229, and 276). These proteins have specific functions in the replication of the viral genome and degradation of the DNA of the bacterial host, and the suppression of the host’s genetic expression by the action of the virus (Murphy, 1998; Sharples et al., 1998). The genome does not contain any conserved antibiotic-resistance genes.

### Lysis module

The Holin sequence (CDS 254) belonged to the bacteriophage T holin family (PF11031), and both lytic proteins (CDS 120 and 154) belonged to the glycoside hydrolase family 24 (PF00959). CDS 154 contained one additional Gp5 N-terminal OB domain (PF06714) and three Gp5 C-terminal repeat domains (PF06715), suggesting a cytoplasmatic lysozyme. SignalP and PrediSi did not identify canonical signal peptides within the lytic protein sequences, and transmembrane-predicting software did not identify transmembrane regions at the N-terminal region of putative endolysins. This analysis shows that the lysozymes encoded in the phage genomes must follow the holin-endolysin theory. Lysozyme hydrolyzes the β-(1,4) linkages between N-acetylglucosamine (NAG) and N-acetylmuramic (NAM) acid monomers of peptidoglycan. Holins control lysis timing by forming non-specific pores in the cytoplasmatic membrane. Finally, the pores allow endolysins to reach the peptidoglycan (Gontijo et al., 2021a).

### Genomic comparisons and pan-genome analysis of *Tequatrovirus*

**Figure 2:**
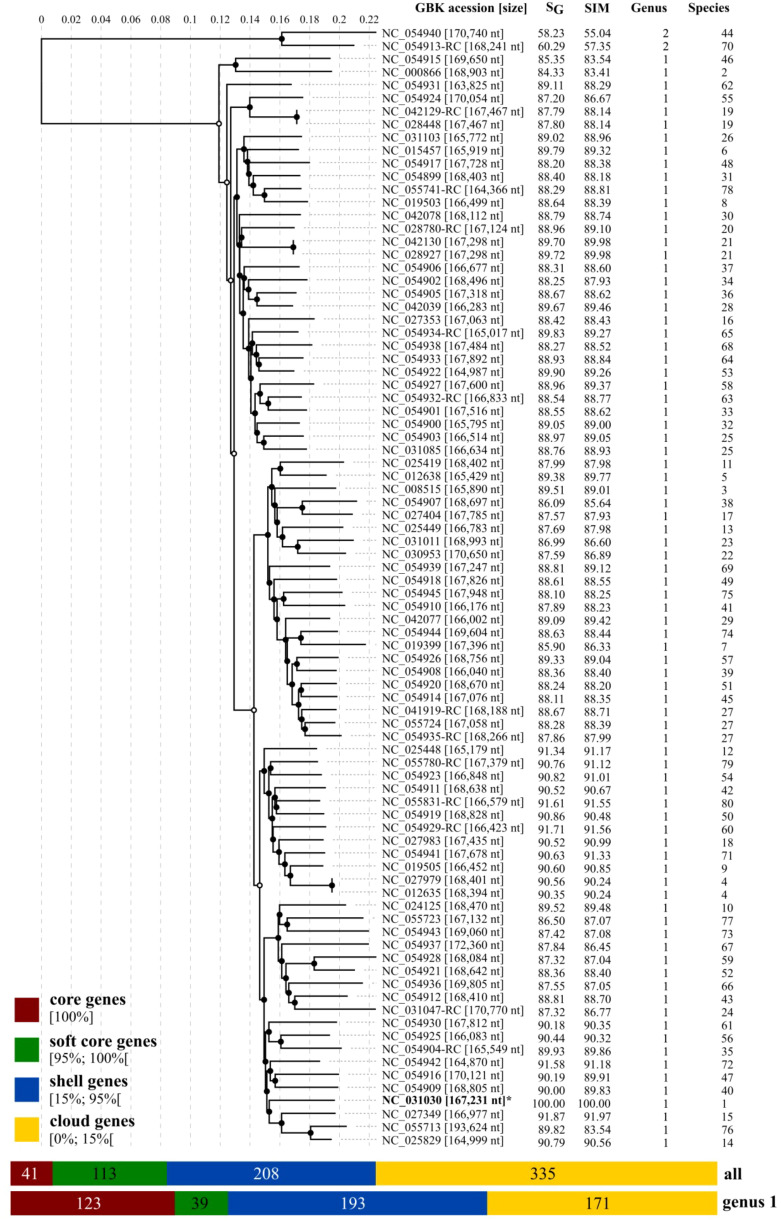
Viral proteomic tree of RefSeq genomes for Tequatrovirus genus. The proteomic tree includes T. ufvareg1 and 84 reference genomes for the Tequatrovirus genus. The genomes were aligned all against all by ViPTree, and the genomic similarity scores (SG) were computed. The genomes were also clustered by VIRIDIC using the genomic similarity (SIM) threshold of 70% for genus assignment (Genus) and 95% for species assignment (Species). The pangenomes were predicted by clustering the protein sequences with an identity threshold of 80%.

The genomic distance matrix (VIRIDIC) and the viral proteomic tree (ViPTree) confirmed high conservation among 83 of the 85 reference genomes of *Tequatovirus* and the genome of *T. ufvareg1* (**Figure 2**, **Table S1**). These genomes showed an average genomic similarity score (VIRIDIC) of 88.77% and a genomic distance score (ViPTree) of 88.91% (**Table S1**). None of the genomes showed similarity scores above 95%, attributing different isolates to the same species, confirming the isolate UFV-AREG1 as a reference for the *Tequatrovirus ufvareg1* specie. Curiously, the genomes of *Tequatrovirus efftwo* (GenBank accession: NC_054913.1) and *Tequatrovirus jayka*y (GenBank accession: NC_054940.1) are divergent from other *Tequatrovirus*. Their genomes showed scores below the 75% recommended by ICTV to cluster species into the same genus. The pangenome analysis confirmed these observations when the number of genes of the core genome of *Tequatrovirus* increased from 41 to 123 genes after removing the genomes of *T. efftwo* and *T. jaykay* from the analysis. Therefore, these two phages represent a distinct genus that is very close evolutionary to the *Tequatrovirus* genus.

**Figure 3:**
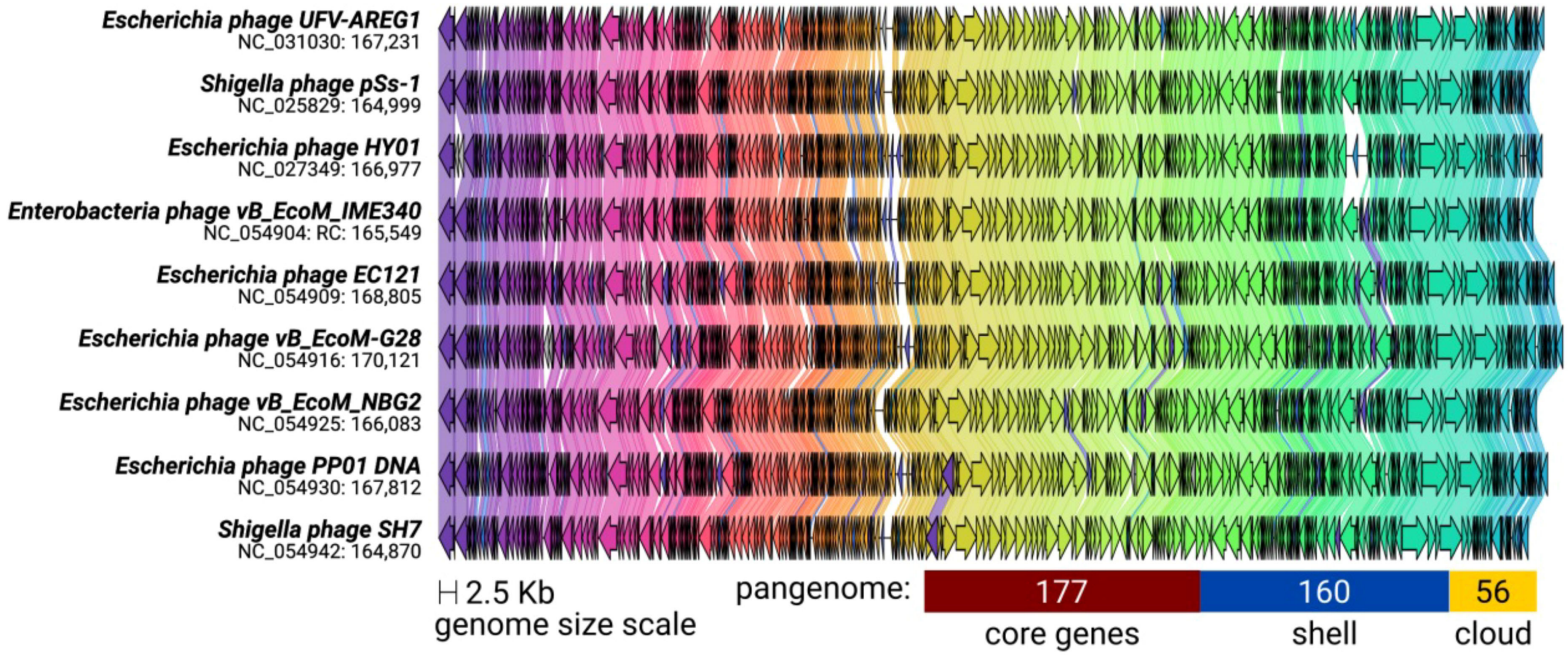
Synteny shared among *Tequatrovirus* genomes clustered with *T. ufvareg1*. The genomes have a bidirectional organization with 267 coding DNA sequences on average (represented by arrows). The arrow’s colors represent the gene clusters identified by Clinker, which encode similar proteins. The lines connecting the arrows represent gene-encoding proteins that share a significant sequence identity of more than 40%. The pangenome was predicted by clustering the protein sequences with an identity threshold of 80%.

The ViPTree included the *T. ufvareg1* into a cluster with eight other *Tequatrovirus* genomes (**Figure 2**). In this cluster, the most similar genomes to *T. ufvareg1* are those from *T. hy01* (*Escherichia* phage HY01) (GenBank accession: NC_027349; VIRIDC score: 91.97%; ViPTree score: 91.87%), *T. sh7* (*Shigella* phage SH7) (GenBank accession: NC_0054942; VIRIDC score: 91.18%; ViPTree score: 91.58%), and *Shigella* phage psSs-1 (GenBank accession: NC_025829; VIRIDC score: 90.56%; ViPTree score: 90.79%). All the eight *Tequatrovirus* genomes clustered with *T. ufvareg1* are colinear (**Figure 3**), and the pangenome analysis confirmed the high conservation by identifying 177 core genes, 160 shell genes, and 56 cloud genes. Core genes are found in >95% of the genomes; shell genes are found in 15–95%, while cloud genes are present in less than 15% of genomes (Page et al., 2015). None of the 268 CDS predicted in *T. ufvareg1* are exclusive genes in pan-genomic analysis, and 13 CDS are cloud genes showing conservation less or equal to 15% among the genomes (**Table 1**). The CDS 93 encodes the ModB ADP-rybosilase, involved in the regulation of the replication cycle; the CDS 24 encodes the Mrh transcription modulator, which modulates the host’s heat shock sigma factor (σ32) (Mosig et al., 1998); the CDS 242 encode the Frd.3 hypothetical protein, and the other 9 CDS encode hypothetical proteins with unknown functions.

**Table 1:** Genes of *Tequatrovirus ufvareg1* genome classified among cloud genes in pangenome analysis.

Gene ID	[locus] (strand)	Annotation	N1	N2
AREG1_00024	[13730:14216] (-)	Mrh transcription modulator under heat shock	12	3
AREG1_00075	[40227:41067] (-)	hypothetical protein	3	1
AREG1_00093	[51717:52203] (-)	ModB ADP-ribosylase	4	2
AREG1_00108	[57796:58009] (-)	hypothetical protein	1	1
AREG1_00129	[67122:67677] (-)	hypothetical protein	2	1
AREG1_00143	[69662:69887] (-)	hypothetical protein	3	1
AREG1_00144	[69955:70264] (-)	hypothetical protein	6	2
AREG1_00145	[70324:70597] (-)	hypothetical protein	4	2
AREG1_00185	[109345:110038] (+)	hypothetical protein	11	3
AREG1_00232	[138074:138785] (-)	hypothetical protein	6	2
AREG1_00242	[143834:144062] (-)	Frd.3 hypothetical protein	5	3
AREG1_00266	[163056:163209] (-)	hypothetical protein	7	1
AREG1_00267	[163289:163463] (-)	hypothetical protein	4	1

N1: Frequency among all Tequatrovirus. N2: Frequency among the T. ufvareg1 cluster.

Cloud genes are those that showed conservation less or equal to 15% among the eight four reference genomes for Tequatrovirus, considering an identity threshold of 80%.

### Host range of *T. ufvareg1*

The host range of *T. ufvareg1* bacteriophage using HostPhinder (Villarroel et al., 2016) revealed that it might be able to infect four bacterial genera: *Escherichia* (E-value: 5.7e-01), *Yersinia* (E-value: 5.5e-01), *Shigella* (E-value: 5.7e-01) and *Salmonella* (E-value: 1.1e-02), all belonging to the *Enterobacteriaceae* family. At the species level, *T. ufvareg1* is predicted to infect *Escherichia coli* (E-value: 5.7e-01), *Shigella flexneri* (E-value: 5.7e-01), *Yersinia pestis* (E-value: 5.5e-01), *Shigella sonnei* (E-value: 1.9e-02) and *Salmonella enterica* (E-value: 1.0e-02). *Tequatrovirus ecomufv133* isolated and propagated using *Escherichia coli* 30 showed similar results after HostPhinder prediction(da Silva Duarte et al., 2018). *In vitro* determination of host, the range was evaluated against *Salmonella* Enteritidis, *Salmonella* Typhi, *Salmonella* Cholerasius, *Escherichia coli*, *Pseudomonas aeruginosa*, *Pseudomonas fluorescens*, *Enterococcus faecium*, *Enterobacter aerogenes*. Lysis was observed only for *E. coli* strains (Lopez et al., 2018a).

## Conclusion

The *de novo* assembled genome of the *Tequatrovirus ufvareg1* bacteriophage isolate has 167,231 bp, a GC content of 35.35%, and 278 predicted genes. The genome annotation is detailed in this study, and 268 CDS encode proteins attributed to eight functional categories. Genomic comparisons confirmed that the UFV-AREG1 isolate only represents the *T. ufvareg1* specie. The analysis of *Tequatrovirus* species revealed high conservation among their genomes and pangenome. The *Tequatrovirus ufvareg1* causes rapid cell lysis liberating about 18 viral particles per infected, indicating the bacteriophage has a high potential as an effective biocontrol agent of *E. coli* O157:H7 in foods.

## Data availability statement

The datasets generate for this study can be found in figshare: https://figshare.com/s/9ef5934fd016b8cb09e8 and will be published under the following: https://doi.org/10.6084/m9.figshare.21696890.

## Author contributions

Conceptualization: ML, ME, DB, and RM; Data curation: ML, MG, and PV; Formal analysis: ML, MG, and PV; Funding acquisition: RM; Investigation: ML, MG, RC, LB, and PV; Methodology: ML, MG, RC, LB, and PV; Project administration: RM; Resources: RM; Software: MG and PV; Supervision: PV, ME, DB, and RM; Validation: MG and PV; Visualization: ML, MG, and PV; Writing – original draft: ML and MG; Writing – review and editing: ME and PV. All authors contributed to the article and approved the submitted version.
