# Supplementary material for: Complete genome analysis of Tequatrovirus ufvareg1, a Tequatrovirus species inhibiting Escherichia coli O157:H7
Source: Front Cell Infect Microbiol. 2023 May 16;13:1178248. doi: 10.3389/fcimb.2023.1178248 (PMC10236363; doi:10.3389/fcimb.2023.1178248)
Supplement: Supplementary file 1 [file DataSheet_1.docx]

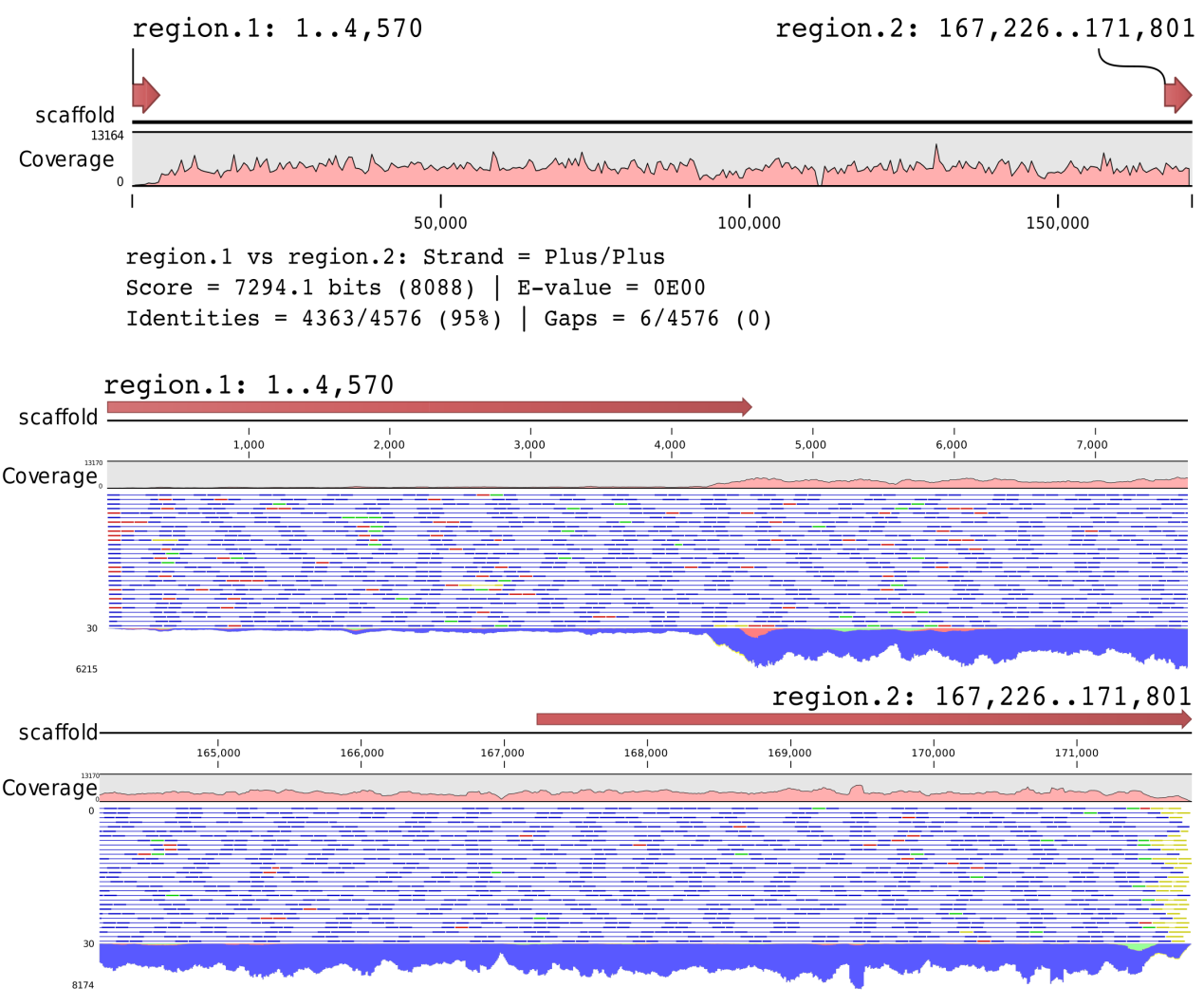


**Figure S1. *De novo a*ssembly of *Tequatrovirus ufvareg1* genome**. The genome was assembled into a contig of 171,801 nt containing highly similar flanking regions of 4,570 nt (region.1 and region.2).
